# Supplementary material for: Versatile roles of rubredoxin reductase of Pseudomonas aeruginosa TBCF10839 in virulence and stress protection
Source: PLoS Pathog. 2025 Sep 3;21(9):e1013465. doi: 10.1371/journal.ppat.1013465 (PMC12431658; doi:10.1371/journal.ppat.1013465)
Supplement: Attachment — Submitted filename: point_to_point_response_PPATHOGENS_reviews_April2025.docx [file ppat.1013465.s005.docx]

PPATHOGENS-D-25-00006
Versatile roles of rubredoxin reductase of Pseudomonas aeruginosa TBCF10839 in virulence and stress protection
PLOS Pathogens

Dear Drs. Priebe, Samuels, Bhaduri-McIntosh and Malim,

First, we would like to thank you and the referees for their time and efforts to review our manuscript mentioned above. In light of your and the reviewers’ valuable and insightful comments, we have revised the manuscript. We have uploaded a clean copy and a marked copy of the revised manuscript.

Please find below our point-to-point response to your recommendations and the reviewers’ comments. For your convenience, we first repeat the reviewers’ statements written in italics followed by our reply written in normal font.

We followed your recommendations:

- Primary data of the PMN phagocytosis assays are shown.
- Likewise, the primary data of H_2_O_2_ dose-response curves are presented.
- As requested, we revisited all figures and legends and edited figure labels and figure legends if applicable.
- PAO gene chip analysis of the mRNA expression profiles is critically discussed. In the light of our current knowledge of the *P. aeruginosa* transcriptome, we describe the limitations of gene chip hybridization and of bulk cDNA sequencing, the latter currently dominating the field.

You addressed the Journal requirements:

- An Author Summary is provided.
- The manuscript does not contain any copyrighted material.
- We confirm that the first author (LW) is the photographer of Figures 1, 3, and 5.

***Reviewers' Comments:*** *Reviewer's Responses to Questions*

***Part I - Summary*** *Please use this section to discuss strengths/weaknesses of study, novelty/significance, general execution and scholarship.*

*Reviewer #1: Wiehlmann and colleagues describe the underappreciated role of rubredoxin reductase (RR) in the virulence of Pseudomonas aeruginosa. Using the CF isolate P. aeruginosa strain TBCF10839, they describe the isolation of a transposon-insertion mutant (rubB::Tn5) with increased susceptibility to neutrophil action. The characterization of this mutant reveals the downregulation of genes and compounds implicated in quorum sensing. They further report increased susceptibility to hydrogen peroxide. The lack of RR also results in decreased virulence in a C. elegans slow-kill assay. The manuscript is well-written and the implication of a role for rubredoxin reductase in P. aeruginosa virulence that goes beyond its canonical function in alkane degradation is intriguing.
However, the data as presented in the manuscript are promising starting points for further investigation but do not always sufficiently back the claims. In some instances, data are not shown. A lack of quantitative rigor for some data sets makes their interpretation difficult. The number of biological and technical replicates should be indicated for all figures.*

We thank reviewer 1 for the insightful evaluation. We apologize that we were a little bit sloppy and were selective in showing the primary data. The number of biological and technical replicates is now indicated for all figures.

*Reviewer #2: This is an interesting study by Wiehlmann and colleagues describing the how rubredoxin reductase has roles beyond its putative function in P. aeruginosa virulence. It opens of several potential lines of investigation and explains one element of how stress responses and regulatory circuits are interrelated in this bacterium.*

Thank you very much for your positive assessment.

*Reviewer #3: The manuscript by Wiehlman et al. deals with the identification of the rubredoxin system in virulence and stress response in Pseudomonas aeruginosa. This is a unique contribution to the field. The study is based on the creation of a genome-wide mutant library and screening for reduced virulence in an in vitro system. The screening identified the rub system as responsible for reduced virulence, which was subsequently restored by trans-complementation.
A curious feature is that the rub system is critical for alkane assimilation via oxidation of alkanes to the corresponding alkanol. Thus, finding that the rub system is involved in redox homeostasis, quorum sensing (QS), and virulence is a very unexpected result.*

We appreciate the reviewer’s statement. Like the reviewer, we were taken by surprise to learn from the screening of the STM library in phagocytosis tests that the rub system is instrumental for strain TBCF10839 to persist in neutrophils. Much to our regret, the IVET and STM approaches are not very popular anymore in our current multi-omics times. Multi-omics generate an avalanche of big data so that one may not see the wood for the trees. Conversely, the screening of STM libraries may identify unexpected targets that are key for the virulence of the microbe in the habitat of interest. However, like a ‘Scarlet Pimpernel’ story, it takes a long time from study design to the final stage that one gets novel insights into virulence and pathogenicity and can make sense of the role of the target in host-pathogen interactions.

***Part II – Major Issues: Key Experiments Required for Acceptance*** *Please use this section to detail the key new experiments or modifications of existing experiments that should be absolutely required to validate study conclusions.

Generally, there should be no more than 3 such required experiments or major modifications for a "Major Revision" recommendation. If more than 3 experiments are necessary to validate the study conclusions, then you are encouraged to recommend "Reject".*

*Reviewer #1: The transposon mutant that has previously been isolated in the neutrophil screen should ideally have been recreated as a clean deletion, given that it is central to almost every experiment. Complementing this mutant is helpful, but data for the complement are not always shown.*

We agree that the comparison of wild type strain, mutant and complemented strain are central for many experiments. To show unequivocal results, the presentation of data is enlarged. First, we now implement the phenotype of the complemented mutant if applicable. Second, since deletions in TBCF10839 do not work reliably in our hands, experiments were validated by assessment of multiple complemented strains. Third, the classical complementation approach may be undermined by putative secondary phenotypes introduced by the copy number of the plasmid complemented in trans. Secondary mutations may have emerged during the handling of the strains. Inspired by the reviewer’s comment, we have sequenced aliquots of stock cultures of wild type TBCF10839 and of the transposon mutant that have been repeatedly used during the course of the study. Moreover, we have determined the copy number in stock cultures of the complemented strain. Fourth and most importantly, the key features of rubredoxin reductase were examined with the purified recombinant protein in defined settings of biochemical assays and fast kinetics. Our manuscript is probably one of the first contributions that combine the plate-based side-by-side comparison of phenotypes of wild-type, mutant and complemented strain with stopped-flow kinetics to resolve the underlying mechanism and reaction kinetics.


*The rubB transposon mutant was identified previously (Wiehlmann et al., JMM 2007). However, in the 2007 publication, I could not find any details on the severity of the survival defect for this mutant in the PMN phagocytosis assay. Given the importance of this mutant for the current study, the authors should provide a quantification of the mutant survival (ideally, of a deletion mutant of rubB) and its complementation (currently, no information is provided regarding whether complementation rescues the transposon mutant in the survival assay.*
The outcome of the PMN phagocytosis assays is now presented in the text and a novel table.

*Figure 1A: Are these spot colonies? Please provide a scale bar. Ideally, this should be presented as a spot dilution assay or liquid culture growth for a more quantitative assessment.*

We apologize that our presentation in the Figure Legend is too concise and that the plate assay has not been described in the Methods section. Figure 1A shows large colonies on an agar plate. The suggested spot dilution assay or the liquid culture growth perform poorly with alkanes. Without an undesirable emulsifier, (extra carbon source!) medium and hydrocarbon will not mix. The unavoidable phase separation in the absence of an emulsifier will stimulate anaerobic growth at the interface. To avoid these confounders, the *P. aeruginosa* strains are inoculated on a 0.2% agar plate that contains mineral medium, but no carbon source. Bacteria are exposed to hexadecane vapor. This reproducible procedure is now described in the Methods section and its principle mentioned in figure legend 1A.

*Figure 1B: Please label the figure. By eye, it is difficult to make a judgment about the coloration of the pellet, especially given the differences in pigmentation of the surrounding culture. Absorption spectra would help compare the strains.*
We now label the color by RGB and provide the quantitative distribution of the signals of the three primary colors as novel information. We agree with the reviewer that a quick look may not discern the differences in pigmentation. Nevertheless, we consider the display of pellets to be the optimal presentation mode. Spectrophotometry is not helpful. Bacteria scatter and thus an absorption spectrum is not reliably resolved in transmission mode. If we lyse the bacteria, the signal is immediately lost because we communicate the redox status of the whole cell. We have asked physicists, engineers, colleagues at our Laser center and renowned experts for optical devices. Nobody knew of a device that records incident / reflected light. Thus, we provide the RGB values. Fortunately, due to the successes of the software we now can provide the quantitative data of the pixels of all three channels. These data are now implemented into Figures 1B, 1C as the quantitative parameter to differentiate the color of the pellets. Moreover, the pellets convey an important message. The knock-out of rubredoxin reductase changes the color of the bacteria highlighting the importance of rubredoxin and rubredoxin reductase for the overall redox status of the cell.

*Figure 2A: It took me a while to understand what I was looking at. Please label the P. putida strain on the plate.*

Following the reviewer’s advice, we labelled the *P. putida* strain.

*Figure 2B and C. It may be sufficient to show Figure 2C.
In figure 2C, the label for the complementation strains obscures where 3-oxo C12-HSL would show up.*
Figures 2B and 2C show two different biosensors that detect C_4_- and C_6_-HSL (B) and 3-oxo-C_6_ and 3-oxo-C_12_ HSLs (C), respectively. Hence, we would like to retain the figures. We consider it the beauty of these figures that they demonstrate the absence of the HSLs in the rub mutant, but similar quantities of the four HSLs in wild type and complemented strain.

We agree with the reviewer that the label for the complemented strain overlays with the 3-oxo-C_12_-HSL signal. Hence, Figure 2C was edited. The photograph was trimmed and labels were located above and below the photograph.

*Figure 2E, F. The legend indicates that the complementation strain TBCF10839 rubB::Tn5; pME6010::TBrubB is shown. But it is not part of the figure.*

Apologies. We indeed only show wild type and mutant. The inclusion of the complemented strain was erroneous. As shown in Figures 2C, 2D, the complemented strain produces homoserine lactones like the wild type. The degradation of elastin in the Congo-red assay is a slow process. As shown in Figures 2E, 2F the major difference between wild type and mutant is seen during the stationary phase in a rather short time window. The assay with the complemented strain did not give consistent results. If we removed tetracycline from the medium, the complemented strain lost the plasmid. Conversely, growth in liquid culture in the presence of tetracycline changed the growth characteristics. Growth of the complemented strain on plates was reproducible, but not the growth in liquid culture.

*Figure 3A. To allow the reader to assess the effect of H2O2 on the three depicted strains, a dose-response curve should be shown for all three strains. This is described in lines 205-211 but the data are not shown.*

The comment is appreciated. We learnt during the revision process that we had used a stock solution in which a substantial portion of H_2_O_2_ had already been degraded when we monitored the dose-response curve. The relative susceptibility of the *P. aeruginosa* strains to the bactericidal H_2_O_2_ was correct, but the estimate of the concentration was wrong. This error within the first version of the manuscript has been eliminated. Our sincere apologies. We are grateful to the reviewer that we were urged to repeat and to expand the experiments. The response of the bacterial strains to H_2_O_2_ is now shown for a liquid and a plate assay. Both procedures yielded consistent results. The dose-response curve of the plate assay with its many concentrations is depicted in the novel Figure 4.

*Line 711-714: The authors write “Within the first 30 seconds P. aeruginosa TBCF10839 (left) reduces hydrogen peroxide to water, whereas TBCF10839 rubB::Tn5 (right) decomposes hydrogen peroxide by disproportionation to produce water and oxygen foam.” Can the authors be certain that TBCF10839 (left) reduces hydrogen peroxide to water in the first 30 seconds? An alternative explanation is that O2 formation is delayed for another reason. Consumption of H2O2 should be monitored to substantiate this claim (e.g. using an assay kit). Please also quantify the data in the supplemental information (bubble formation). How many replicates were performed?*

The reviewer cites a phrase from the legend of Figure 3B. Figure 3B shows the phenotype of the acute exposure of *P. aeruginosa* strains to hydrogen peroxide. We agree with the reviewer that the molecular etiology cannot be resolved from this gross assay. We need the combination of Figure 3, the video in the supplement and the stopped-flow kinetic experiments to make the point that the rub system is key to delay the emergence of oxygen bubbles. In all peroxidases and catalases the reaction with hydrogen peroxide will first generate a water molecule and a high-valent iron intermediate, known as Compound I (Cpd I) characterized by an oxoferryl porphyrin cation radical or an electromer of this (Groves et al. JACS 1981;103:2884-2886). It is the pertinent role of the rub system to reduce Cpd I for having a continued production of water molecules. Thus, as long as NADH is available to reduce the rub system, the reaction of Cpd I with a second molecule of hydrogen peroxide is delayed which generates O_2_ and a water molecule. To demonstrate this mechanism, we set up the video with rubredoxin reductase, rubredoxin and catalase in the presence or absence of NADH. In the absence of NADH, oxygen bubbles immediately emerge after the addition of hydrogen peroxide, whereas the pre-incubation with NADH will delay the production of oxygen. The reviewer’s suggestion to monitor the consumption of hydrogen peroxide by the bacteria with an assay kit is not feasible. The manual handling of bacteria and the pipetting of hydrogen peroxide are too slow to cope with these rapid reactions. Hence, to demonstrate the kinetics of the underlying reactions we set up the reconstituted system with the pure compounds to show in a video the production of oxygen bubbles in presence and absence of NADH in real time. The reviewer will agree that the reduction of the rub system by NADH is key to delay the catalase-mediated production of oxygen. In other words, as long as the rub system can reduce CpdI, the catalase behaves as a reducing agent to reduce hydrogen peroxide to water. The reviewer asks us for the quantitation of bubble formation. However, such a counting of bubbles would be semi quantitative at best. Optics and temporal resolution of our device are not suited to determine the volume of the gaseous bubble. By 2025, we can indeed resolve the kinetics of aquatic bubbles (for example, sweat secretion on a human skin monitored online with a USB microscope having a resolution of 1600x1200 pixels), but this approach is not feasible with a gas. Just by serendipity, we have insight into the challenging task of the quantitation of bubbles. It took our colleague Manuel Nietert many months of dedicated programming until voxels of aquatic bubbles could be converted to nanoliter volumes (Supplement in Pallenberg et al, J Cyst Fibros 2022;21,442-447). However, the existing software can only handle the emergence of bubble on planes, but not in 3-D volumes. In other words, bubble quantitation is an inappropriate endeavor. We therefore went one-step further and performed reaction kinetics in stopped-flow experiments that generate high quality robust and reliable quantitative data. Finally, the reviewer asks for the number of replicates of the reactions shown in the video. We have to admit that we did not took notes of all our experiments to include them into the lab book. Nevertheless, we are certain that these experiments have been performed at least 50 times generating always the same outcome.

*Figure 6: The authors demonstrated the effect of the mutation on QS, which is likely to explain the fast-kill phenotype in C. elegans. Whether this is related to the proposed activity of the rubredoxin reductase in H2O2 protection is not addressed.*

Thank you for your comment. We agree with the reviewer that neither the data in the figure nor the text link the findings with any molecular etiology. We have just selected the *C. elegans* model to demonstrate the impact of an intact rub system on the pathogenicity of *P. aeruginosa.* Why the nematode *C. elegans*? Since the late 1990s, *P. aeruginosa* killing of *C. elegans* has been used to identify *P. aeruginosa* virulence factors, even at a genome-wide level (see for example Tan et al. PNAS 1999;96,715-720; Mahajan-Miklos et al. Cell 1999;96,47-56; Tan et al. PNAS 2009;96,2408-2413; Garvis et al. PLoS Pathog 2009;5,e1000540; Feinbaum et al. PLoS Pathog 2012;8,e1002813). Thus, the *C. elegans* infection models were incorporated into the portfolio of the current reference monograph for the methodology of Pseudomonas research (Pseudomonas Methods and Protocols, Methods in Molecular Biology, vol 1149). In other words, *C. elegans* has become a standard non-mammalian infection model in the Pseudomonas field. Our experiments now demonstrate that the rub system is as relevant as the many other well-known pathogenicity factors. We clarify the objectives by an extension of the first phrase in the paragraph: “…we tested whether the pathogenicity of TBCF10839 was attenuated in the AHL-deficient *rubB* mutant using a *C. elegans* infection model *that has become standard to identify virulence factors of P. aeruginosa.”*

*Reviewer #2: There is one significant issue:

1. The transcriptomic analysis is strange. The authors determine gene expression in TBCF10839 (and the rubB transposon mutant) to PAO1 using a gene chip. This approach excludes genes specific to TBCF10839 and potentially those genes that do not have 100% identity between the two strains. Also, chip technology has not been the standard for determination of transcriptomes for over a decade.
I don't believe that the transcriptome needs to be re-done but there are several caveats that must be applied to the current approach, which should be included in the text.*

The reviewer’s comment is very welcome to let us explain why we allocated such a strong role to the gene chip experiments both in a quantitative and qualitative manner.

We will elaborate in more depth on transcriptomics, which may be of general interest for the editors and the reviewers to appreciate the pros and cons of gene chip hybridization and cDNA sequencing in the light of our current knowledge in 2025 of gene expression of *P. aeruginosa*. When you have read our response, you hopefully will not qualify anymore our ‘transcriptome analysis as strange’.

a. A PAO1 chip does not represent the TBCF10839 genome

First, we fully agree with the reviewer that the gene chip just covers the core genome but not the accessory genome of the TBCF10839 strain. However, both PAO1 (6,264,403 bp) and TBCF10839 (6,915,509 bp) belong to the ExoS clade, thus the genome-wide nucleotide sequence diversity between the two strains just accounts to 0.37% among the shared genes (6.1 Mbp) and hence does not affect the hybridization of TBCF10839 cDNA onto the gene chip. The PAO1 and TBCF10839 sequences of *rubB* are 99.63% identical.

b. The impact of the PAO1 chip data on our research journey.

Within the Pseudomonas field, the authors’ group (LW & BT) belonged to one of the first teams to use the PAO1 gene chip to study core genome-wide gene expression profiles of *P. aeruginosa* strains (Microbiology 2004;150,831-841). Later, within the Pseudomonas community we were the first group to compare gene chip hybridization with strand-specific cDNA sequencing by second generation sequencing technology (Environ Microbiol 2011;13:1309-1326) and we were also the first group to compare direct RNA Nanopore sequencing of the *P. aeruginosa* transcriptome with that of cDNA sequencing (J Bacteriol 2022;204:e0041821).

Thereby we learnt during the last 25 years

- that the evaluation of gene expression by rank number from highest to lowest level provided similar datasets for Affymetrix microarrays and cDNA sequencing
- that the fold- changes and their distribution for significantly differentially regulated genes were similar for gene chips and cDNA sequencing
- that the oligonucleotide hybridization techniques failed to distinguish expression levels within the groups of very lowly and very highly expressed transcripts because of unfavorable background noise or signal saturation, respectively (a trivial, but important point)
- that cDNA sequencing detected differentially expressed genes at the same frequency at all expression levels ranging from the most to the least abundant transcripts
- that in the case of the Affymetrix microarrays the frequency of significantly differentially expressed genes linearly declined with decreasing absolute expression level
- that cDNA datasets published by our peers have often been evaluated with insufficient stringency (by loving eye Benjamini-Hochberg corrections)
- that the commonly applied high-throughput sequencing of size-selected cDNA fragment libraries of reverse-transcribed RNA preparations is burdened with quite some severe flaws: The depletion of rRNAs, enzymatic reverse transcription, and the fragmentation, size selection, and amplification during library preparation lead to inevitable losses of information about the initial composition of the RNA pool.
- that the commonly analyzed bulk transcriptome differs substantially from the primary transcriptome of the same RNA preparation
- that the cDNA transcriptome enriched for primary transcripts matches with the nanopore RNA transcriptome
- that almost all published cDNA datasets of *P. aeruginosa* transcriptomes missed the large repertoire of antisense RNAs.

In summary, we admit that the hybridization of gene chips has limitations. However, the published transcriptomes based on bulk cDNA sequencing are also burdened by a similar level of limitations (that the respective authors typically did not address).

Now, we would like to stick to show the gene chip data in the manuscript. Why?

First, during the last 20 years, we reported PAO1 gene chip hybridizations in nine papers, seven of which included strain TBCF10839. In five papers, the transcriptome of the wild type strain was compared with that of transposon mutants of the STM TBCF10839 library. The possibility to compare PAO1 chip datasets of several TBCF10839 transposon mutants provides added value to this manuscript.

Second, we combined the four-comparison survival method with the criterion of the Bonferroni-corrected 95% confidence interval. This stringent approach generates robust, reproducible and reliable datasets of differentially expressed genes that will sustain the test of time. (Unfortunately, the evaluation of most cDNA sequence datasets of the *P. aeruginosa* transcriptome lack this stringency.)

Third, we were taken by surprise that so many key genes of metabolism and signaling were downregulated in the TBCF10839 *rubB*::Tn*5* mutant by more than seven-fold. Even applying our very stringent criteria, we detected a large set of core genes that encode well-known and highly relevant tasks in the bacterial cell. Referring to reviewer 1, the unexpected gene chip data of differentially expressed genes initiated our long-lasting research journey to explore ‘the underappreciated role of rubredoxin reductase in the metabolism and virulence of *P. aeruginosa*‘. Thus, the Results section starts with the unexpected insights into the *P. aeruginosa* transcriptome of TBCF10839 *rubB*::Tn*5* that demonstrates the lack of so many key features of the ordinary *P. aeruginosa* cell. Even though Table 1 is very long, we would like to retain the gene chip hybridization data in the main body of the manuscript.

To cope with the reviewer’s comment, we included the information that PAO1 gene chips ‘cover 87.7% of the 6.9 Mbp large TBCF10839 genome with 99.63% nucleotide sequence identity’. The fact that our approach only tackles the core genome has repeatedly mentioned in the first version of the manuscript and thus the wording is retained.

*Reviewer #3: None*

Thank you.

***Part III – Minor Issues: Editorial and Data Presentation Modifications*** *Please use this section for editorial suggestions as well as relatively minor modifications of existing data that would enhance clarity.*

*Reviewer #1: Please use consistent nomenclature in the figures and text for the mutant and complementation strains.*

Thank you for your comment. We learnt that we were indeed a little bit sloppy in the nomenclature of mutant and complementation strains. Wording was changed if applicable.

*Reviewer #2: 1. Line 145 - the table of genes that were upregulated might be moved to a supplement so the reader can focus on the down regulated genes.*

We admit that the Table is very long. However, as outlined in our elaborate reply to reviewer 2’s general criticism to use PAO1 Gene Chips for transcriptome analysis, we applied very stringent criteria to identify the differentially regulated genes. Just focusing on the rather small set of genes whose expression was differentially regulated by 7-fold or more, the text describes the black-and-white picture of up- and downregulated gens in TBCF10839 *rubB*::Tn*5.* The mutant upregulated the expression of rmf and downregulated key genes of metabolism, signaling and virulence. Hence, we would like to retain Table 1 as it is.

*2. Line 152 - las is not an operon*

Thank you for this important comment. The error was corrected.

*3. Figures 2BC are difficult to interpret.*

We do understand the reviewer’s judgment that one has to look carefully to grasp the message of the photographs that TBCF10839 *rubB*::Tn*5* does not secrete AHLs. Conversely, wild type and complemented strains secrete similar amounts of C_4_-, C_6_-, 3-oxo-C_6_ and 3-oxo-C_12_ HSLs. Psychologically, quick readers will overlook an empty lane even if this finding conveys the key message. To channel the message to the reader, we included the interpretation of the photographs into the Figure Legend.

*4. Figure 6. These data are probably best presented in a K-M survival plot, and should be appropriately statistically analysed.*

We appreciate the comment. The figure has been redrawn and the data were evaluated by Kaplan-Meier Survival Analysis.

*An additional note: I appreciated the video in the supplement!*

Thank you.

Reviewer #3: Suggestions for improvement:

• *The Introduction focuses heavily on the alk system and alkane oxidation, but other aspects of Pseudomonas biology are not well addressed. I suggest the authors begin the Introduction by highlighting commonalities between environmental isolates of Pseudomonas aeruginosa and clinical strains. This could include shared carbon source utilization pathways and virulence factors. Then, the authors can describe the rub system as previously reported in the context of alkane degradation.*

We cordially thank the reviewer for this comment to reconsider and expand the introduction. Considering the recently published structural biology work, the introduction has just dealt with alkane oxidation that so far has been the only topic where rubredoxin reductase and rubredoxin seemed to have a role for *P. aeruginosa*. Encouraged by the reviewer’s suggestion, we now start the introduction with a paragraph on the major features of the lifestyle of *P. aeruginosa* in disease and environment.

*• In general, avoid the use of abbreviations to start paragraphs or subsection headings.*

Thank you for your comment. Abbreviations were removed from subsection headings.

*• Line 129: The comparison between 67 vs. 59 genes is unclear—please clarify.*

The term ‘thereof’ apparently sounded ambiguous. As requested, we clarified the phrase.

• *Table 1 is organized by PA gene number. It may be more useful to group the genes by function as described in the text. This would help make the long table more digestible.*

We understand the reviewer’s intention to make Table 1 more digestible. As already mentioned above, we admit that Table 1 is very long even though we restricted the display to genes that are more than 7-fold differentially regulated. Many genes encode multiple functions. When the *P. aeruginosa* PAO1 genome was published in 2000, it was helpful that PAO1 ORFs were assigned to one of 25 functional categories derived from those used for classification of *E. coli* ORFs. However, by 2025 we have learnt from thousands of curated GO terms and manually-curated annotation updates of the Pseudomonas Community Annotation Project that we should stick to the organization of the Table by PA gene number to sustain the test of time. The reviewer may check the Gene Ontology section of Pseudomonas Genome Database to appreciate the multiple functions that are assigned by 2025 to a *P. aeruginosa* ORF. Moreover, please note that by May 2025 still 2129 of 5570 ORFs are annotated as ‘hypotheticals’ of unknown function.

*• Since rubredoxin may interact with multiple proteins, some docking assays could be considered (not necessarily required for this manuscript).*

As shown in the global impact of the rubredoxin reductase – rubredoxin complex on the overall redox state of the *P. aeruginosa* population (see Figures 1B, 1C), rubredoxin needs to interact with many proteins. Docking assays are a good idea, but we are afraid to say that due to its general importance of RubR – RubA2 for the homeostasis of the cellular redox status the outcome of the experimental enterprise will likely be more a catalogue or even an encyclopedia of all iron centers involved in redox reactions. Rubredoxin is a phylogenetically ancient protein. To demonstrate this ability of the NADH –RubR –RubA2 electron transfer system to reduce any iron center, we made a case with horse cytochrome C (see Fig. 5 in the manuscript).

• *The potential of rubredoxin as a target for anti-Pseudomonas therapies could be an interesting point to explore in the discussion.*
Thank you for bringing up this inspiring idea. As addressed in the previous comment, the RubR/RubA2 complex interacts with many targets and thereby governs the redox status of the *P. aeruginosa* cell. Rubredoxins are present in a few clades of bacteria and archaea, but also in cyanobacteria, red algae, cryptophytes, green algae and plants. Thylakoid-membrane-bound homologs of soluble RubA2 perform oxygenic photosynthesis. Plant-associated rubredoxins have a rubredoxin domain fused to a C-terminal transmembrane helix that is absent in soluble bacterial rubredoxins such as *P. aeruginosa* RubA2. Nevertheless, the inspection of the tree of life teaches us that the phylogenetically ancient bacterial rubredoxin is not an appropriate target for anti-Pseudomonas therapies because of its substantial homology with the orthologs of photosystem II derived from endosymbiosis of a cyanobacterium. Photosystem II (PSII) drives oxygenic photosynthesis and produces most oxygen present in the Earth’s atmosphere. The redox-active rubredoxin domain of chloroplast-located rubredoxin 1 protects PSII intermediate complexes from photooxidative damage, which may explain why it is essential in photosynthetic eukaryotes. Hence, we refrain from any discussion of *P. aeruginosa* RubA2 as a target for antimicrobial therapy.

*• Line 190: “Transfected” should be replaced with “transformed.”*
corrected as requested

*• Was the whole genome of the rub mutant sequenced?*
Inspired by the reviewer’s query, we have taken aliquots from the stock cultures of the wild type and of the TBCF10839 *rubB*::Tn*5* mutant that had been repeatedly used during the course of the study and then have performed ultra-deep sequencing of the genomes. Compared to the PacBio-based reference sequence of TBCF10839 that we had deposited at NCBI, two sequence variants were detected in the coding region (one synonymous and one non-synonymous nucleotide substitution) in the four genome sequences. This information is now reported in the novel paragraph ‘Genome sequencing’ and the novel Table S2 in the Results section. Moreover, we identified pairs of co-existing sequence variants showing the nucleotide of the deposited TBCF10839 sequence and another nucleotide. About 80% of the nucleotide pairs are G-C or C-G transversions, are located in the non-coding region and predominantly segregate in four 2- to 20-bp large hotspots (cf. Table S2). We observed an overlapping spectrum of co-existing nucleotide pairs in the wild type and the transposon mutant. When the mutagenesis was performed, the transposon should have inserted in a genomic background devoid of any co-existing nucleotide pairs. Hence, apparently the very same nucleotide substitutions occurred in the mutant after the insertion of the transposon. This serendipity is fascinating in the context of molecular evolution (and according to our knowledge has yet not been reported for *P. aeruginosa*), but this topic of course distracts from the objectives of this study. The findings are summarized in the novel paragraph ‘Genome Sequencing’ at the end of the Results section.

*• Define in the text the selectable marker carried by plasmid pME6010.*

The shuttle vector pME6010 (GenBank accession no. AF118810) contains the ‘RK2 tetracycline resistance protein’ TetA and the ‘RK2 tetracycline repressor protein’ TetR of the Birmingham-group IncP-1*α* conjugative plasmid RK2 isolated from *Klebsiella aerogenes* K8841 as selectable markers. This information has been included in the text.

**Figures**

We have uploaded the figure files to the Preflight Analysis and Conversion Engine (PACE) digital diagnostic tool. PACE did not report any Image Problems or PACE Adjustments. Uploaded and PACE-processed versions are identical implying that the figures comply with the demands of the Journal.

That’s all. We are grateful to the editors and reviewers for their instructive and inspiring comments and ideas. Hopefully you will enjoy the revised version of our manuscript.

We look forward to hearing from you.

Kind regards on behalf of all authors

Burkhard Tümmler, MD PhD
