# Supplementary material for: Versatile roles of rubredoxin reductase of Pseudomonas aeruginosa TBCF10839 in virulence and stress protection
Source: PLoS Pathog. 2025 Sep 3;21(9):e1013465. doi: 10.1371/journal.ppat.1013465 (PMC12431658; doi:10.1371/journal.ppat.1013465)
Supplement: Attachment — Submitted filename: Response_to Reviewers_Aug2025_second_revision.docx [file ppat.1013465.s006.docx]

PPATHOGENS-D-25-00006R1
Versatile roles of rubredoxin reductase of Pseudomonas aeruginosa TBCF10839 in virulence and stress protection

Dear editors,

Thank you for the thoughtful evaluation of our revised manuscript. Reviewers 2 and 3 had no comments, but you and reviewer 1 addressed several points that are dealt with below. For your convenience, we first repeat the comment (written in italics) followed by our reply.

First, you specified your request:

*In addition to a point-by-point response to comments of Reviewer 1, focusing on the rigor of quantification and statistical analyses, we ask you to elaborate on the curve fit methodology and to provide statistical analysis of colony size on hydrogen peroxide (Fig. 4); you will likely need to consult with a statistician regarding the appropriate test to use (which will likely require curve-fitting, regression and root mean square deviation; a two-way ANOVA is not suitable).*

You ask for a novel statistical analysis of the colony size depicted in Figure 4. You recommend to consult a statistician to address this non-trivial task. Figure 4 and its legend show the primary data and two trend lines. We appreciate the critique that the statistical analysis should be improved: A more appropriate statistical approach to perform a proper regression of this kind of dataset requires machine learning with a kernel so that the datasets of wild type and mutant are separated and a linear regression analysis becomes feasible. However, to apply the kernel, we first would like to address the experimental background of the generated data followed by our conclusion.

As written in the legend of Figure 4, wild type and mutant strain were grown in LB and then pelleted by centrifugation. The pellet was suspended in LB adjusted to an OD (600 nm) of 0.05. Next, ten µl were manually dropped on agar plates containing 1 to 32 mM hydrogen peroxide. The drop will contain a spatially rather homogeneous suspension of bacteria. However, the contact area of the agar surface and the pressure applied by the pipet were not standardized (which inherently is not feasible by manual pipetting onto the surface of a jelly-like mass). These minute drop-by-drop variations typically do not influence the quality of the primary data, but they are critical in our setting. The distribution of bacteria in the center or at the border of the contact area will make a difference for the survival of the bacteria exposed to a sub-lethal concentration of hydrogen peroxide. As you can deduce from our video, rubredoxin/rubredoxin-reductase is critical for the survival of the bacteria during the first minute of the exposure to hydrogen peroxide until the expression of catalase, superoxide dismutase, alkylhydroperoxide reductase and thioredoxin reductase 2 is upregulated by 10- to 200-fold (Salunkhe P et al. J Bacteriol. 2005;187:2565-72). Correspondingly, as you can see in Figure 4, the colony size of the *P. aeruginosa* mutant that lacks a functional rubredoxin/rubredoxin-reductase, drops within a narrow range of hydrogen peroxide concentration from the normal range to zero. On the other hand, in case of the wild type strain with a proficient rubredoxin/rubredoxin-reductase, we observed a broad range of the individual colony size over a broad range of the concentration of hydrogen peroxide. Thus, Figure 4 sends the message that the survival of wild type and mutant are strongly distinct upon exposure to hydrogen peroxide. Nevertheless, taking the inevitable drop-by-drop variation into account that is formally, but in our eyes not unequivocally healed by a kernel, we decided to repeat the analysis from scratch with two other complementary approaches.

First, in line with the reviewer’s request referring to Figure 3A, bacterial inocula (OD_600 nm_ = 0.05 equivalent to about 10^8^ CFU/ml) of wild type strain and mutant were grown in LB during exposure to a series of ten two-fold dilutions of hydrogen peroxide covering the concentration range of 0.25 mM to 128 mM. Second, taking the same precultures, about 100 bacteria each were plated onto a series of agar plates of two-fold dilutions of initial concentrations of 3.2 mM or 2.4 mM hydrogen peroxide. These two experimental approaches substitute colony size as endpoint. The new datasets are now presented in the novel Figure 4 backed up by adequate statistics that are more straightforward and more robust than a kernel. Both datasets demonstrate the higher resistance of the RbR proficient strain to hydrogen peroxide, but they also reiterate the common knowledge that isolated bacteria are substantially more susceptible to a toxic agent than bacterial communities are.

In summary, we are grateful to the editors and reviewer 1 to urge us to substitute the assessment of colony size as an endpoint by two more robust and complementary quantitative approaches.

*Reviewer #1: line 115: The authors state that cultures reached early stationary phase after 20 h of growth in LB. This seems slow, given that under typical batch-culture conditions (with shaking) P. aeruginosa PAO1 and PA14 usually reach early stationary phase after 5 h of growth. It would be helpful to see growth curves. The timing of growth phases can sometimes be inaccurately estimated if growth curves are not plotted on a logarithmic scale.
Figure 2E and F:
It is still not clear to me why a difference between the two strains is only observed at ~24-28 hours.*

*how many data points are averaged in the respective graphs?
please connect data points with straight lines*

We would like to combine our answers to these two comments. The reviewer is wondering about our statement that our cultures reached early stationary phase after 20 h of growth. According to our experience *P. aeruginosa* PAO1 and PA14 have left the mid-exponential phase after 5 h of growth under typical batch-culture conditions. By this time point, the bacteria have still not reached the early stationary phase, but by 10 h a growth curve will typically reach its plateau. We agree with the reviewer that the time point of 20 h is late. Thus, we re-investigated bacterial growth in different growth media and culturing conditions, particularly proper aeration. Thereby we indeed had to recognize that the growth of our *P. aeruginosa* strains was variable and that the culturing conditions significantly influenced the growth curve. Hence, we agree with the reviewer that under improved growth conditions the bacterial strains reached the stationary phase after about 10 h of culture.

In our context, we focus on the onset of quorum sensing during the stationary growth phase as the reference point. The secretion of elastase is taken as the appropriate surrogate parameter. LasB and to lesser extent LasA are unique among *P. aeruginosa* proteases in their ability to degrade elastin. As you can see for the standard elastin Congo-Red assay, the degradation of elastin increases for a few hours until the PQS system takes over. Please note that the *N*-acyl homoserine-lactones (AHLs) triggered the increase of elastin proteolysis within this time window for the AHL-proficient wild type and complemented strains, but not for the AHL-negative transposon mutant. This difference disappears when gene expression is governed by the PQS system during the late stationary phase.

In summary, we focus on the onset of quorum sensing during stationary phase. We agree with the reviewer that in our hands the OD will indeed not increase anymore by the 10 h time point. We apologize for the confusion that our writing may have caused. Hence, to eliminate this confusion we now chose a time-independent presentation of the primary data, which demonstrates the onset of quorum sensing during stationary phase as a function of optical density. Thus, Figures 2E and 2F are substituted by a single Figure 2E, whereby each point represents the mean of five independent measurements.

*Figure 1B, C: Please label x- and y-axes for the RGB plots. They may be better visible on a white background.*
The label needs to be legible. The five rectangles are small in size. Hence, we constructed one common coordinate system and labelled the axes with a legible font size. Further, the meaning of the axes are explained in the figure legend.

*Figure 3A:
Instead of showing a single image, it would be preferable if the author showed growth curves integrating multiple data sets.*

Thank you for your suggestion. However, within the reasoning and the flow of thoughts of the chapter ‘Rubredoxin reductase – rubredoxin confer oxidative stress protection‘ we would like to stick to the presentation of single representative images in Figure 3A. First, we set the stage by showing the data of phagocytosis assays with human neutrophils. The *rubB* transposon mutant is impaired in its intracellular persistence in neutrophils. Thus, the hypothesis arises that the mutant is less fit to cope with hydrogen peroxide, which is a major antimicrobial weapon of neutrophils. The hypothesis is then tested by related assays with wild type, mutant and complemented strain always applying the same protocol for the preparation of the bacterial inoculum:

A	survival of bacteria after short-term 5 min exposure to a lethal concentration of hydrogen peroxide

B	long-term growth of bacteria in a medium with an initial concentration of 10 mM hydrogen peroxide (Figure 3A)

C	when exposed for 30 s with a lethal concentration hydrogen peroxide, the wild type strain reduces hydrogen peroxide to water, whereas the *rubB*::Tn*5* mutant decomposes hydrogen peroxide by disproportionation to produce water and oxygen foam (Figure 3B)

D	Video: kinetics of the production of oxygen foam by catalase upon exposure to 15 mM hydrogen peroxide in the presence of rubredoxin reductase and rubredoxin A2 ± 3.3 mM NADH.

E	novel information implemented into the second revision: Monitoring growth of wild type and mutant strain in liquid medium during exposure to initial concentrations of 0.25 to 128 mM hydrogen peroxide (Figure 4A) (in line with the reviewer’s request to show growth curves)

F	novel information implemented into the second revision: CFU titration of the survival of wild type and mutant strain growing on LB agar containing various concentrations of hydrogen peroxide within the range of 1 to 32 mM (Figure 4B)

G	Demonstration of the underlying reaction mechanism: Stopped flow kinetics of the transfer of reduction equivalents from NADH in bulk solution to the FAD prosthetic group of *P. aeruginosa* rubredoxin reductase followed by the transfer of reduction equivalents of the FADH_2_ intermediate of RubR to *P. aeruginosa* rubredoxin.

Please note that at the chosen initial concentration of 10 mM H_2_O_2_ there is an absolute dichotomy of growth of TBCF10839 (normal bacterial density), TBCF10839 *rubB*::Tn*5* (no growth) and TBCF10839 *rubB*::Tn*5*; pME6010::TB*rubB* (normal bacterial density). Thus, a photo is the adequate medium to transmit this information. As suggested by reviewer 1, growth curves were implemented (see point E).

*Figure 4:
How was the trendline generated? Please indicate the average colony sizes with dedicated symbols and connect them with straight lines.
What do the indicated symbols represent? I assume they are the averages of three technical replicates?*

Figure 4 with its data on colony size is substituted by a novel Figure 4 that displays CFU titrations and growth curves. For more information about background and approach, please read our response to the editors’ request at the beginning of our rebuttal letter. Your comment was implemented into the editors’ request.

*line 263: The authors conclude that the rubredoxin system rescues the bacteria from H2O2 exposure. There may indeed be a modest effect. However, given the spread of data points its difficult to infer if it is statistically significant. Please include a statistical analysis.*

Again, please read our response to the editors’ request at the beginning of our rebuttal letter. Your comment was implemented into the editors’ request.

That’s all.

We thoroughly thank reviewer 1 and the editors for their comments and suggestions. You urged us to substantially improve the quality of our manuscript by substituting Figure 4 by two novel datasets.

We look forward to hearing from you.

Kind regards

Lutz Wiehlmann

Burkhard Tümmler
